# Psychiatry in the sultanate of Oman

**DOI:** 10.1192/bji.2023.24

**Published:** 2023-11

**Authors:** Hamed Al-Sinawi, Hassan Mirza

**Affiliations:** 1 Senior Consultant Psychiatrist, Department of Behavioural Medicine, Sultan Qaboos University Hospital, Muscat, Oman; 2 Senior Consultant Psychiatrist, Department of Behavioural Medicine, Sultan Qaboos University Hospital, Muscat, Oman. Email mirza@squ.edu.om

**Keywords:** Oman, psychiatry, mental health services, mental disorders

## Abstract

Oman has been recognised as the leading country in terms of recent developments in public health. However, there is a shortage of mental health services, which poses challenges for people seeking such services, particularly those from rural areas. This often results in delayed treatment and longer periods of untreated mental illness.

The Sultanate of Oman is the 69th largest nation in the world and occupies a 309 500 km^2^ territory south-east of the Arabian Peninsula.^1^ Oman has benefited from higher living standards as a nation with a relatively high income since the 1980s.^2^ According to the National Centre for Statistics and Information, Oman's population touched the landmark 5 million figure for the first time in 2023;^3^ 58% of this population comprises Omani citizens and almost 65% of the total are young people under 29 years of age.^4^ The number of young people in Oman has recently increased and this trend is anticipated to remain for the next 20 years. Hence, Oman will face difficulties in the next 20 years in meeting the demands of the growing number of young people, particularly in providing quality education, healthcare and future employment possibilities.^5^ The capital Muscat is the largest city in Oman, where most of the population resides, and the only city with tertiary care mental health facilities.

## Mental health services in Oman

The public health system of Oman is accessible free of charge to its citizens from the moment they are born until the time of their death.^6^ As a result, Oman was ranked number 1 out of 191 WHO member states for its overall performance on the level of health by the World Health Organization (WHO) in its first-ever comparative examination of health systems in 2000. In addition, Oman was ranked first among the top 10 nations worldwide for recent advancements in public health in the United Nations’ 2010 Human Development Report.^7^ Nevertheless, psychiatric services are scarce by and large, making it difficult for people to access mental health facilities, especially people from rural areas compared with those living in urban areas. This leads to a delay in treatment initiation and, hence, a longer duration of untreated mental disorders.^8^ Oman's Ministry of Health (MoH) provides most of the country's medical services. The overall number of psychiatrists employed by the MoH is 60, and a further 13 psychiatrists are employed by other institutions.^9,10^ Also, there are 17 psychologists and 28 social workers employed by government institutions in Oman.

In the capital city of Muscat, two large hospitals – the psychiatric ward of Sultan Qaboos University Hospital and the Al Masarra Hospital – provide most in-patient mental health treatment.^9^ Similarly, Sultan Qaboos Hospital in Salalah, southern Oman, has a small in-patient facility. The College of Medicine and Health Sciences is affiliated with the teaching hospital Sultan Qaboos University Hospital. Patients in any region of Oman can receive tertiary care there. The in-patient unit has 28 psychiatric beds for people of both genders. The service offers many subspecialties, including general adult psychiatry (in-patient and out-patient), child and adolescent psychiatry, geriatric psychiatry, consultation-liaison psychiatry and neurology. The Al-Masarra Hospital, established in 2013, is a 245-bed tertiary care psychiatric facility staffed by multidisciplinary teams that include psychiatrists, doctors, dentists, psychiatric nurses, pharmacists, psychologists, social workers, occupational therapists, physiotherapists and speech therapists. In addition, the hospital offers a variety of mental health treatments, including consultation-liaison psychiatry, geriatric medicine, addiction services, child and adolescent mental health and general adult psychiatry, including integrated services for forensic psychiatry.^11^ Concerning Oman's human resources in mental healthcare, there are 5.8 psychiatric beds, 1.54 psychiatrists and 10.3 nurses per 100 000 population.^9^

## Child and adolescent mental health services

In the late 1990s, the Sultan Qaboos University Hospital launched the first child and adolescent mental health (CAMH) service, which provided basic services.^12^ This service has lately developed a more thorough and multidisciplinary strategy. It is the first psychiatric in-patient unit for young people in Oman, and owing to high demand, beds are accessible for urgent admissions 24 h a day, 7 days a week. It seeks to provide comprehensive care for children and young people with mental illnesses who are under the age of 18. The service includes both out-patient and in-patient management, with the latter being a unique programme that requires a caregiver to be an attendant on the ward with the patient throughout the in-patient stay. Similarly, Al-Massara Hospital offers comprehensive CAMH services. However, the ethical conundrum in treating children and adolescents, such as contentious mandatory admission and involuntary treatment procedures, is a significant difficulty facing child and adolescent psychiatrists in Oman.^13^

## Geriatric psychiatry

The mental health service for older adults was established in Oman in 2011 at Sultan Qaboos University Hospital. The service provides healthcare to patients from all over Oman, including memory and neuropsychological assessment. The number of patients gradually expanded as the team conducted regular training workshops for primary healthcare doctors on assessing people with cognitive impairment, which increased the referrals of these patients to the memory clinic. The team also runs public awareness programmes about Alzheimer's and other dementias.^9^ A similar service catering to the mental health needs of older adults has been established at Al-Massara Hospital, offering in-patient care and a limited community service within the capital city.

## Undergraduate education

The Sultan Qaboos University's general medical training programme lasts 7 years (4 years preclinical, 3 years clinical). Graduates rotate through three of the four specialties during their 1-year internship (internal medicine, general surgery, child health, obstetrics and gynaecology).^14^

For Omani trainees to understand the social and cultural elements of illness and well-being, behavioural science – which spans various disciplines – is taught in the second and third years of the preclinical course.

The first time clinical students encounter psychiatry is during an 8-week clinical clerkship in the subject during their sixth year. The 8-week curriculum comprises additional lectures on various facets of clinical psychiatry and related subjects, patient case presentations, case histories and clinical interviews.^9^

## Postgraduate education

The Oman Medical Specialty Board is the postgraduate medical training body in Oman and it was established in 2006. It has adopted the Accreditation Council for Graduate Medical Education International competencies (ACGME-I) to achieve excellence in postgraduate medical education, training, assessment and accreditation throughout the Sultanate of Oman.^15^ The psychiatry residency training programme granted ACGME-I accreditation in 2017 is a 5-year programme designed to produce specialists in general psychiatry with adequate knowledge and competency in the subspecialties. As part of this rotation, the residents rotate between general adult, old age, child and adolescent, addiction and consultation-liaison psychiatry, among other psychiatric subspecialties. In addition, before being awarded a specialty certificate in psychiatry, residents must complete the monthly in-training evaluations and pass the mandatory written and clinical examinations.

The postgraduate trainees are eligible for a scholarship to complete a 2-year clinical fellowship abroad in a field of their choice at a reputable training facility which is ACGME-I approved.^16^ There have been 75 graduates of the psychiatry programme since the postgraduate training programme's commencement, with an average of 6 residents completing the residency programme each year.

## Challenges and future perspectives

Since their beginning in the early 1980s, mental health services in Oman have had difficulty growing and they are still in their infancy. Whether mental health services are prepared to handle the problems of the coming 10–20 years is unknown. An increasing and ageing population in Oman will undoubtedly strain the country's mental health facilities further. Other factors include the lack of integrated services, budget restrictions and workforce development.

Moreover, there has been little to no development in community mental healthcare, which has led to a rise in the number of individuals becoming institutionalised for long periods in psychiatric facilities. Such obvious deficiencies will promote stigma and make it impossible for patients to live independently in the community.

Similarly, Oman is currently dealing with a rise in drug misuse. The proximity of Oman to the main routes used to traffic opioids presents a significant problem for the country's addiction treatment programmes. According to research by Narconon International, drug overdose deaths and drug-related crimes are rising in Oman.^17^ As a result, a substance misuse unit has been established in the country's newly constructed psychiatric hospital. Despite this, substance misuse in Oman is still a complicated problem with many contributing factors.^18^

Moreover, scarce resources and minimal government expenditure on mental health mean that services remain below the recommended requirements for a better quality of life.^19^ According to cultural and religious beliefs, a sizable proportion of Muslim Arabs still thinks that the evil eye, magic and jinn possession are the causes of mental illnesses.^20^ Another common characteristic in the Arab world is the reliance on a deity and religious figures to deal with mental health difficulties.^21^

In this situation, many Omani families turn to traditional healers before contacting mental health specialists, and this is still a substantial societal barrier to Omanis using mental health treatments.^22^

Overall, the absence of mental health legislation in Oman has not hindered the approach of multidisciplinary teams to involving patients and their families, despite the shortcomings experienced by mental health professionals dealing with psychiatric patients. Oman strives to provide comprehensive care for people with mental illnesses, with in-patient services available round the clock for urgent admissions, a growing community service and a mental health act in the pipeline.^13^

## Data Availability

Data availability is not applicable to this article as no new data were created or analysed in this study.
